# Distributed Fiber Optic Shape Sensing of Concrete Structures

**DOI:** 10.3390/s21186098

**Published:** 2021-09-11

**Authors:** Christoph M. Monsberger, Werner Lienhart

**Affiliations:** 1 Institute of Engineering Geodesy and Measurement Systems, Graz University of Technology, Steyrergasse 30/II, 8010 Graz, Austria; werner.lienhart@tugraz.at; 2 ACI Monitoring GmbH, Ragnitzstrasse 163/2, 8047 Graz, Austria

**Keywords:** distributed fiber optic sensors, shape sensing, concrete structures, civil structural health monitoring, beam load testing, tunnel lining segments

## Abstract

Civil structural health monitoring (CSHM) has become significantly more important within the last decades due to rapidly growing construction volume worldwide as well as aging infrastructure and longer service lifetimes of the structures. The utilization of distributed fiber optic sensing (DFOS) allows the assessment of strain and temperature distributions continuously along the installed sensing fiber and is widely used for testing of concrete structures to detect and quantify local deficiencies like cracks. Relations to the curvature and bending behavior are however mostly excluded. This paper presents a comprehensive study of different approaches for distributed fiber optic shape sensing of concrete structures. Different DFOS sensors and installation techniques were tested within load tests of concrete beams as well as real-scale tunnel lining segments, where the installations were interrogated using fully-distributed sensing units as well as by fiber Bragg grating interrogators. The results point out significant deviations between the capabilities of the different sensing systems, but demonstrate that DFOS can enable highly reliable shape sensing of concrete structures, if the system is appropriately designed depending on the CSHM application.

## 1. Motivation

Aging infrastructure in combination with rapidly growing construction volume worldwide involves challenging conditions for civil engineering structures. Appropriate designs as well as suitable constructions are therefore indispensable to ensure safe construction works and reliable operations. In this context, structural health monitoring (SHM) becomes a powerful procedure to analyze the structure’s integrity and to predict the prospective behavior for maintenance works. Adequate sensor selection and placement represent one of the key factors to develop a cost-efficient and, finally, successful SHM approach. The variety of conventionally used methodologies range from electro-optical and electro-mechanical sensors to acoustic emission, fiber optics, remote sensing, and imaging techniques, as well as vibration measurements [1].

Distributed fiber optic sensing (DFOS) has significantly evolved in recent years to monitor large scale civil infrastructures, such as bridges [2,3,4,5], high-rise buildings [6], reinforced earth structures [7], pipelines [8,9], or tunnels [10,11,12,13]. The technology enables distributed, continuous strain and temperature measurements with high accuracy and high spatial resolution over kilometers, where the sensing fiber can be directly embedded inside the structure. Beside the detection of damages and failures like cracks or leakages, the deformation development over time can also be utilized for condition-based maintenance.

Especially in concrete engineering, the identification and localization of local deficiencies is essential to evaluate the integrity of the structure. DFOS is advantageous compared to traditional embedded sensors as the structural behavior can be continuously assessed as a whole without any gaps between the sensing points. A wide range of laboratory studies on crack detection and localization using DFOS along concrete beam structures is known in literature. These discuss different sensor installation techniques, such as bonding along the beam’s surface using various sensing fibers [14] and adhesives [15], mounting cables inside the concrete [16], or gluing in grooves along reinforcement bars [17]. The resulting DFOS strain profiles can either be utilized to calibrate developed strain transfer models [18,19], to derive effective crack widths along the structure [20,21] or to establish alert levels for SHM applications [22].

The evaluation of curvature and bending characteristics along civil infrastructure can supply beneficial information to compare the actual condition, including, for instance, damages and fatigue, to the construction and planning state. Investigations already demonstrated that long gauge strain sensors are suitable for strain-based shape sensing [23,24], if the sensors are appropriately arranged along the structure and the loading scenario is known. Recent studies also discuss the embodiment of fiber Bragg grating (FBG) sensors, e.g., into 3D printed patches [25], for high-resolution curvature sensing at discrete locations. DFOS, however, enables new capabilities for in situ shape sensing inside concrete due to the fully-distributed sensing feature in combination with high spatial resolution. Deflection profiles can be derived without detailed knowledge of the loading situation, if two or more boundary conditions are known [26].

This paper introduces a comprehensive analysis of distributed fiber optic shape sensing of concrete structures. The study includes detailed investigations on various concrete beams using different DFOS sensors and installation techniques, as well as monitoring of real-scale tunnel lining segments. The installations were interrogated by fully-distributed sensing units based on Rayleigh and Brillouin scattering as well as by quasi-distributed FBG interrogators. In the following, basic characteristics of DFOS systems for civil engineering applications are reviewed (Section 2) and the shape-sensing algorithm, including an analysis of the DFOS’s spatial resolution impact supported by laboratory investigations, is introduced (Section 3). Results of different load tests are presented and verified using, inter alia, pointwise displacement readings and distributed image-based measurements (Section 4 and Section 5). Finally, the outcomes are concluded and an outlook on practical SHM applications is given (Section 6).

## 2. Distributed Fiber Optic Sensing in Civil Engineering

Distributed and quasi-distributed fiber optic sensors have become significantly more popular in structural and geotechnical applications as they can provide high-resolution measurements from the inside of the structure. For this, however, the fiber optic interrogation unit, the sensing cables, or the FBG sensors, respectively, and the installation technique must be appropriately adjusted to ensure the quality of the measurement results.

### 2.1. Sensing Principles

Fiber optic sensors have already been used over the past decades in several scientific fields. In civil engineering applications, commonly utilized sensor types can be divided into (i) fully-distributed sensors, (ii) quasi-distributed FBG sensors [27], and (iii) pointwise interferometric sensors (cf. SOFO sensor [28]). The latter two will not be further discussed in this paper due to the focus on distributed applications.

**Figure 1:**
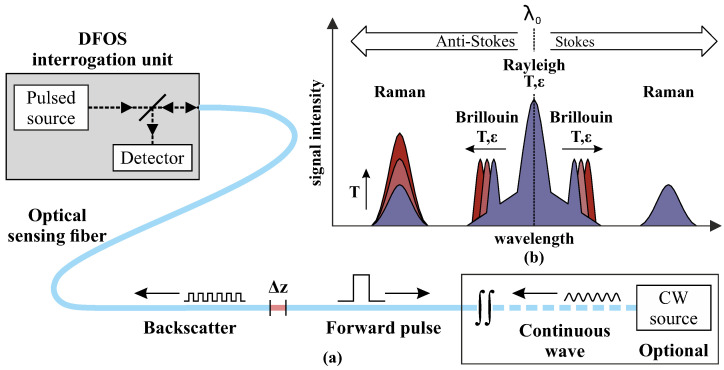
Distributed fiber optic sensing techniques [30]: (**a**) Basic scheme of sensing setup. (**b**) Different scattering components in optical glass fibers.

Fully-distributed fiber optic sensing systems use the natural scattering of optical signals during the forward propagation along the sensing fiber (Figure 1a). Small parts of these intensity losses are backscattering effects, whose spectral characteristics carry information about geometrical, physical, or chemical quantities. The backscattered spectrum can be split into linear (Rayleigh) and nonlinear (Brillouin and Raman) scattering, see Figure 1b. Raman systems are sensitive to temperature only, whereas Rayleigh and Brillouin instruments are sensitive to both strain and temperature changes [29]. Their capabilities regarding spatial resolution, repeatability and measurement duration are however significantly different as listed in Table 1. It must be highlighted that these indicated specifications are general values, which are varying depending on the manufacturer, the DFOS cable, and the sensing parameters.

Rayleigh scattering based on the Optical Frequency Domain Reflectometry (OFDR) technique enables single-ended measurements with a high spatial resolution and measurement precision, but these measurements are limited in the sensing range. Brillouin sensing units are capable to measure over long distances, which however results in spatial limitations and typically, longer measurement times of several minutes. This sensing technology must be further divided into systems using a loop setup (Brillouin Optical Time/Frequency Domain Analyzer) or a single-ended configuration (Brillouin Optical Time/Frequency Domain Reflectometry), which essentially restricts the measurement repeatability. The selection of the appropriate DFOS technologies, therefore, always involves a trade-off between the sensing range, spatial resolution, and strain sensing precision, which must be suitably adjusted depending on the requirements of the actual civil engineering application.

### 2.2. DFOS Cables

Sensing along or inside civil infrastructure objects always implies harsh environment for sensors. Therefore, an appropriate protection of the sensitive element combined with a reliable stress transfer from the structure to the sensor is essential to realize successful monitoring approaches. Optical fibers are generally advantageous for sensing as they are lightweight, flexible, and can be easily attached to the structure. Nevertheless, the glass material itself is fragile and might be more susceptible for potential damages, which is why the sensing fibers must be protected using additional protection layers depending on the civil engineering application.

**Figure 2:**
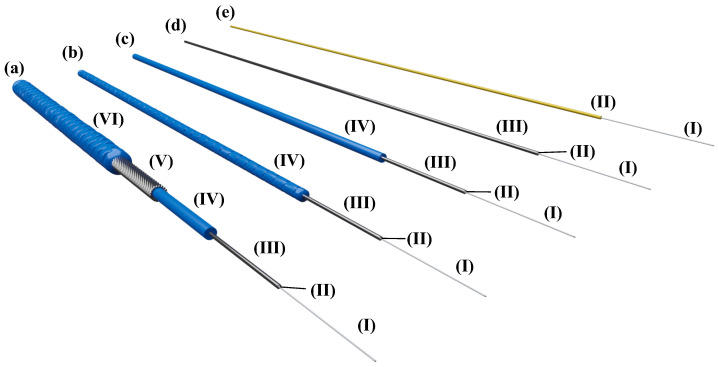
Distributed fiber optic sensing cables for application along concrete structures with (I) strain sensing single mode fiber (⌀ 250 μm), (II) tight buffer, (III) metal tube, (IV) polyamide protection layer, (V) special steel armoring, and (VI) polyamide outer sheath: (**a**) BRUsens V3. (**b**) BRUsens V9. (**c**) BRUsens V4. (**d**) BRUsens FiMT. (**e**) Tight-buffered fiber.

Commercial suppliers offer a wide range of DFOS cables with different setups, well designed for specific applications in different fields. Figure 2a–d depicts a selection of strain sensing cables from Solifos AG (Switzerland), which are specially developed for sensing in structural and geotechnical engineering. These cables protect the optical sensing fiber through a metal tube, a polyamide sheath, or even a special steel armoring to ensure the sensor’s integrity during installation and monitoring. All cable layers are interlocking for a reliable transfer of mechanical strains to the sensitive glass fiber core. The outer sheath of the cables can be structured to guarantee a solid bond with the surrounding material, cf. grout or concrete. Further information on the cable designs may be gathered from the corresponding datasheets, see, e.g., in [40,41]. DFOS cables with protection layers might not be flexible enough for surface application, especially in case of a tight cable routing. Tight buffered fibers (Figure 2e), normally used in telecommunication industries (see, e.g., in [42]), can overcome this restriction, but are limited in harsh environment due to their fragility.

External temperature changes do not only result in thermal variations of the structure, but also alter the fiber optic strain readings. Therefore, an additional sensing fiber, which is not influenced by mechanically induced strain and, therefore, either embedded within the same cable [43] or loosely installed within a separate cable [44], shall be installed in practical applications next to the strain cable to compensate temperature effects and evaluate the actual stress behavior of the monitored structure.

Newly constructed civil infrastructure is often designed for service lifetimes of up to 150 years [45]. The longevity of the distributed fiber optic sensor inside or along the concrete structure is therefore essential as replacements after the installation entail large efforts or are even not possible. Nevertheless, any durability assessment of DFOS cables is challenging due to lacking knowledge on the long-term stability and missing standards. Investigations on the optical fiber itself demonstrated that the failure probability is always related to the applied tensile stress as well as the bending radius. Installation lengths shorter than 1 km are designed to enable a success rate of about 99% for tensile stresses of up to 800 MPa (=approximately 1% of strain) over 25 years [46]. This time frame might be significantly extended in case of lower mechanical strains, which is why, for instance, Corning Inc. (Somerville, MA, USA) underlines that there is no “theoretical lifetime” for optical fibers [47].

The fiber’s lifespan, however, not only depends on the mechanical properties, but also on external influencing factors, e.g., extreme temperatures or presence of hydrogen. The latter may cause major attenuations along the fiber over time, which restrict the lifetime within long-term monitoring. Here, sensing cable layers (cf. Figure 2a–c) can provide a certain degree of protection for a wide range of applications, especially for transportation infrastructure like bridges or tunnels. For harsher environments, extensive aging tests by the French national radioactive waste management agency (ANDRA) showed that specially carbon-coated fibers are able to withstand high hydrogen and even radiation exposure [48]. From this, it can be concluded that the literature does not provide general guidelines on the long-term durability of DFOS cables. These must be appropriately selected within the monitoring design depending on the requirements on flexibility, mechanical protection, and prevalent monitoring environment.

### 2.3. Sensor Installation Techniques

Besides the selection of the physical DFOS components, the sensor attachment along the monitoring object is critical to guarantee the strain transfer from the structure to the sensing cable. The stress behavior inside civil engineering structures usually varies depending on material properties, loading conditions, etc. Therefore, the sensors must be placed not only at applicable locations, but also using the suitable installation technique with respect to the project requirements.

**Figure 3:**
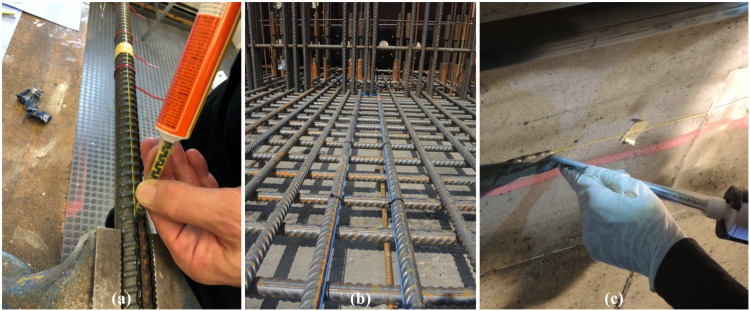
Sensor installation techniques: (**a**) Application along reinforcement. (**b**) Installation inside concrete. (**c**) Application along surface.

For concrete structures, applications inside the structure as well as along the surface are practicable. The monitoring results (further discussed in Section 4 and Section 5), however, significantly vary depending on the installation location and the used DFOS cable type. Reinforced concrete objects enable the direct sensor application inside the structure, where either bare fibers or tiny cable types (cf. Figure 2d,e) may be glued by appropriate adhesives (Figure 3a) on the steal surface or inside grooves to assess the steal strain behavior. More robust sensing cables (cf. Figure 2a–c) can be attached to the reinforcement bars using cable ties (Figure 3b) to capture the concrete capacity. The reinforcement itself also involves the advantage that the cable is better protected during the casting process, especially in case of high concrete pumping pressure.

DFOS surface applications in civil engineering are particularly beneficial for subsequent instrumentation and monitoring. Flexible, e.g., tight buffered sensing fibers can be individually guided along the surface for an areal coverage of conspicuous regions like surface crack areas or similar. As depicted in Figure 3c, the fiber can be directly glued on the surface using adhesive mortars, which also protect the optical fiber against mechanical impacts and provide higher robustness in practical environment.

The sensor installation technique not only affects the monitoring capabilities, but might also be limited because of practical circumstances on-site. The implementation of the sensor must fit to the construction process itself and can be restricted due to lack of time for the installation or access obstacles. Therefore, an appropriate coordination with the construction contractor is indispensable to realize a successful sensor installation.

## 3. Distributed Fiber Optic Shape Sensing Approach

**Figure 4:**
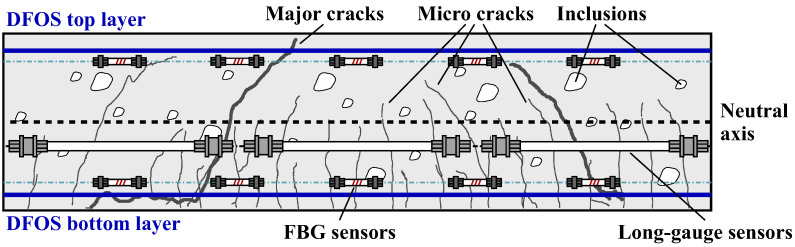
Shape sensing along concrete structures (based on the work in [23]).

Concrete structures in civil engineering are usually affected by local inhomogeneities like inclusions or cracks, which result from the construction process itself or loads during operation. The strain sensor location inside the concrete, as well as the corresponding gauge length, is therefore essential for data interpretation, cf. Figure 4. Long-gauge strain sensors are able to capture the overall behavior of the structure, but are limited to identify local deficiencies. FBG sensors along the structure or point sensors with smaller gauge length can provide the local strain behavior at selected locations, however, with the drawback that events between the sensitive elements might be overlooked. The distributed sensing feature of DFOS systems basically enables both, the comprehensive, gapless acquisition of the strain behavior and the localization of local defects. Nevertheless, the strain sensing results strongly depend on the sensor’s characteristics like position, cable type or interrogation unit, see Section 4 and Section 5.

Fiber optic sensors basically deliver strain (and temperature) values along the longitudinal axis of the installed sensors. If two ore more sensing lines are placed inside the structure in the same geometry in a well-known arrangement (cf. Figure 4), the strain values can be utilized to derive curvature characteristics along the object, which arise due to potential bending orthogonal to the sensing direction.

Based on elastic bending theory, see, e.g., in [49], the deflection *w* at one specific sensing location *s* along an object can be described by

(1) $$w\left(s\right)=\int \int \frac{M(s)}{E\cdot I_{y}}=\int \int \kappa (s)$$

where *M* is the bending moment, *E* the modulus of elasticity, and $I_{y}$ the moment of inertia at the considered position. The deflection *w* can also be expressed by the local curvature value κ and, therefore, by the bending radius *R*. On this point, the relation between the bending radius and the measured strains along the different layers in combination with the distance between the sensors *d* can be used to directly assess the curvature characteristics:

(2) $$\kappa \left(s\right)=\frac{1}{R(s)}=\frac{\epsilon_{top}\left(s\right)-\epsilon_{bottom}\left(s\right)}{d(s)}$$

Beside influencing shear stresses, the concrete object might also be affected by longitudinal stresses due to shrinkage, creepage, or temperature-induced expansion. These effects are taken into account by the longitudinal strain, which is equal to the mean strain value of both sensing layers $\bar{\epsilon }$:

(3) $$\kappa \left(s\right)=\frac{\epsilon_{top}\left(s\right)-\epsilon_{bottom}\left(s\right)}{d\left(s\right)\cdot \left(1+\bar{\epsilon }\left(s\right)\right)}$$

(4) $$\bar{\epsilon }=\frac{1}{2}\cdot \left(\epsilon_{top}\left(s\right)+\epsilon_{bottom}\left(s\right)\right)$$

The curvature determination can be basically carried out without any knowledge of the object’s material properties. However, it is assumed that the individual shape of the cross section between both sensing elements remain constant based on the Bernoulli hypothesis. This might especially be crucial for aging infrastructure or concrete structures with high degree of damage, where systematic deviations can arise in the curvature derivation since the assumption can only be partially fulfilled.

The derived curvature values can be split into segments with length ds and double integrated to evaluate the distributed bending behavior along the object. Numerical integration techniques range from methods based on difference equations [50] to trapezoidal and rectangle rule integration [51]. The latter two are mostly used for pointwise sensing techniques, e.g., long gauge sensors, where the number of sensors along the structure is limited and the boundary conditions are well defined. The difference equation method (DEM) enables a more flexible definition to solve the boundary-value problem using different supporting points along the object. This can be especially advantageous if more than two supporting points can be utilized to provide redundancy and handling of potential erroneous data in practical applications on-site. Using DFOS in combination with pointwise displacement readings, e.g., from total station measurements, can provide an integrated approach, which allows fully distributed shape assessment along the civil engineering structures [52].

### 3.1. Impact of Spatial Resolution

Using pointwise or quasi-distributed sensing techniques, the sensor positions can be well related to specific locations along the object, where the measurement values are captured with a defined gauge length. DFOS allows the distributed and continuous assessment of the structure’s overall behavior without any gaps. Nevertheless, the resulting strain-sensing profiles represent an integrative response within the spatial resolution of the DFOS system and local stress events, e.g., due to structural damages or abrupt changes of the bending moment, might get smoothed. The system’s capabilities with respect to the spatial resolution must therefore be well considered, especially for shape sensing approaches, where an appropriate relation between different sensing layers is essential to provide accurate curvature values (cf. Equation (2)).

**Figure 5:**
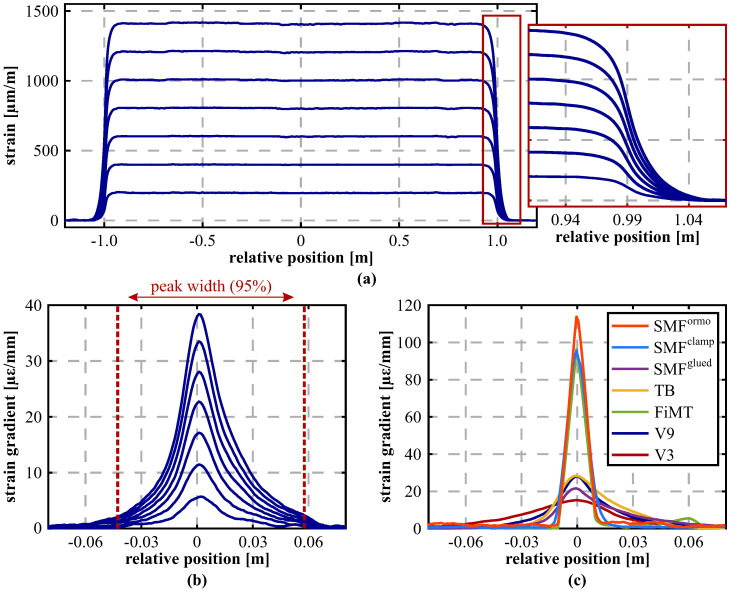
Analysis of spatial resolution impact: (**a**) Distributed strain profiles along strained section of sensing cable BRUsens V9. (**b**) Strain gradient at different load levels. (**c**) Strain gradient for different fiber types at 1000 με.

The spatial resolution of the DFOS signal varies depending not only on the characteristics of the interrogation unit (cf. Table 1) itself, but also on the used sensing cable setup. Various enveloping layers result in different strain transfers from the sensing cable’s outside to the sensitive glass fiber core and, thus, the spatial recording of abrupt strain changes along the fiber is affected. In order to analyze this spatial resolution impact for different sensing fibers and cables, numerous samples with lengths of ~2 m were investigated at different load levels at the IGMS (Institute of Engineering Geodesy and Measurement Systems, Graz University of Technology) strain calibration facility [53] and interrogated using the OBR 4600 from Luna Innovations Inc. (USA) based on the OFDR technique, providing a spatial resolution of 10 mm (Figure 5a). The strain application theoretically causes a discontinuous function at the sample edges, which is smoothed depending on the fiber packing. The influencing area can be evaluated by e.g., determining the strain gradient along the edge section (Figure 5b), which delivers the differential strain change over length. Figure 5c depicts the strain gradient profiles for all investigated cable types at a load level 1000 μm/m (= με). As expected, single-mode bare fibers with standard acrylate and ormocer coating present the most feasible response to the applied strain with a total impact length lower than 3 cm. It is remarkable that the fiber in metal tube (FiMT) shows comparable results, which indicates a reliable bond between the sensing fiber and the surrounding metal sheet. In contrast, sensing cables (V3 and V9) as well as the tight-buffered fiber (TB) deliver much longer impact areas of up to 10 cm or even more.

The resulting strain gradient peak may also be classified to find the numerical peak width at a specific significance level (here it is 95%, cf. Figure 5b). This finally enables the determination of comparable spatial resolution impact lengths for different samples at different load levels as shown in Table 2. Here, it is notable that the strain level itself does not significantly influence the derived impact length, which seems to be almost independent from the applied load. Nevertheless, the attaching method majorly affect the strain response. Whereas the first SMF (SMF 28e, clamped) was clamped at both edges of the testing facility, another sample of the same fiber (SMF 28e, glued) was fixed using an appropriate adhesive. The reduced strain transfer, obviously resulting from the elastic behavior of the glue, causes major differences in the strain gradient behavior (see Figure 5c) as well as in the numerically derived peak width. Beside the interrogation unit’s capabilities and cable structure itself, the characteristics of the attaching method must therefore be also well considered within the DFOS design and data interpretation.

### 3.2. Loading Scenarios

Spatial resolution limitations and corresponding smoothing of strain profiles do not only affect the detection of local stress events, but also the accurate derivation of the curvature profiles. DFOS shape-sensing approaches usually use a model-free displacement profile determination, and therefore substantial knowledge of spatial limitations within derivation procedure is essential.

**Figure 6:**
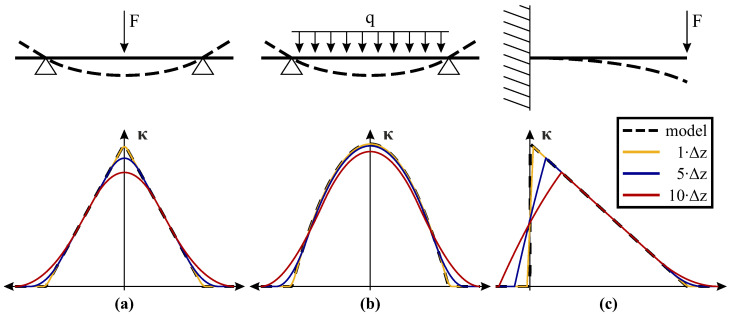
Theoretical analysis of spatial resolution impact on curvature determination: (**a**) Simply supported —single load (bi-linear distribution). (**b**) Simply supported —uniformly distributed load (parabolic distribution). (**c**) Cantilever —single load (linear distribution).

Figure 6 presents three of the most typical loading scenarios of concrete beams in civil engineering and a corresponding analysis of the impact of different spatial resolutions (Δz) on the curvature distribution. Major deviations to the theoretical model resulting from spatial limitations become visible at abrupt changes of the curvature profiles, which can be well observed along the cantilever beam (Figure 6c). Except for these locations, the loading situations can be appropriately captured despite spatial limitations. This suggests that also DFOS approaches with typically lower resolution based on Brillouin scattering can enable a sufficient derivation of displacement profiles.

### 3.3. Laboratory Testing

**Figure 7:**
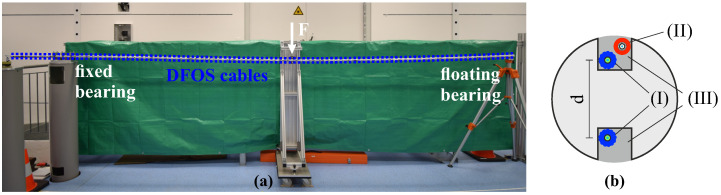
Laboratory testing of steel rod specimen [55]: (**a**) Practical test setup. (**b**) Installation scheme with (I) strain sensing cable, (II) temperature sensing cable, and (III) gluing groove.

To verify the theoretical analysis and to evaluate the feasibility of the shape sensing approach for DFOS systems with different spatial capabilities, a steel rod specimen with a total length of 6 m and a diameter of 30 mm was instrumented using strain sensing fibers in two different layers along the top and bottom side of the object, see Figure 7. The design is optimized to determine vertical bending only. An appropriate arrangement of three sensing fiber layers (e.g., 8, 12, 4 o’clock) would, however, also enable the derivation of 3D bending curves, which is not the focus of this investigation. The specimen itself was modified before the installation with milling grooves (depth of 7 mm) in order to guarantee an exact relative alignment of the sensing layers (Figure 7b). The DFOS strain-sensing cables [41] were glued along the grooves using a two-component epoxy especially fabricated for metal adhesion [54]. Testing was performed at the IGMS laboratory, which is fully air-conditioned at a temperature of 20 °C ± 0.5 °C and a humidity of 50% rH ± 10%, and therefore thermal-dependent length changes can be excluded over the testing period. Nevertheless, an additional temperature sensing cable [44] was installed along one layer to eliminate potential temperature effects in practical applications [55].

**Figure 8:**
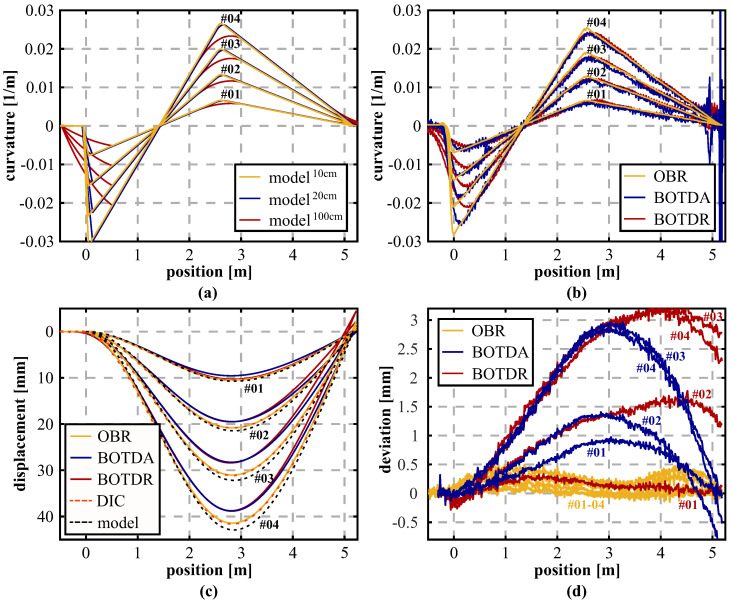
Laboratory testing results: (**a**) Theoretical curvature distribution. (**b**) Curvature profiles derived from DFOS strain measurements. (**c**) Displacement curves calculated from DFOS strains, DIC, and theoretical model. (**d**) Deviations between DFOS and DIC.

The specimen was one-ended fixed on a survey pillar and supported by a floating bearing at the other side, see Figure 7a. Distance-based loading was performed by a screw threads apparatus in the middle of the specimen in equidistant load steps in three full cycles and controlled using a manual dial gauge with a resolution of 0.01 mm. The setup itself realizes a simple support loading scenario in combination with a cantilever, which can also be identified within the theoretical curvature distributions in Figure 8a. The system was subsequently interrogated at each load step using different sensing units based on Rayleigh (OBR, spatial resolution of 1 cm) as well as Brillouin scattering (BOTDA/BOTDR) with a spatial resolution of 20 or 100 cm (cf. Table 1). Theoretical models were evaluated based on these corresponding resolutions, except for the OBR, whose spatial capabilities were set to 10 cm with respect to the outcomes of the BRUsens V9 spatial resolution analysis in Section 3.1. The curvature profiles derived from the DFOS strain measurements (Figure 8b) depict a good agreement with the theoretical analysis for all considered load steps and the loading scenario itself can be well identified with the measured data. The theoretical BOTDR model simulated with a spatial resolution of 100 cm might be even too pessimistic since the curvature curves seem to coincide well with BOTDA measurements excluding the fixed bearing area.

Subsequently, the distributed displacement curves can be determined for each sensing technology. Boundary conditions for the double integration process are defined based on the cantilever approximation, which implies that the starting point at the fixed bearing and its orientation is fixed and, therefore, the determination principle is not controlled at any other point along the object (e.g., at the floating bearing). This enables an analysis of the different spatial limitations as well as their implications over the object length. The resulting displacement profiles in Figure 7c show that the actual deformation behavior is displayed by all sensing technologies and the equidistant load increase is well represented. Nevertheless, the Brillouin scattering techniques display essentially higher systematic deviations to the theoretical bending model, which likely result from the lower spatial resolution and limited measurement precision. Remaining deviations between the high-resolution OBR measurements and the theoretical model might result from uncertainties in the material properties (e.g., modulus of elasticity) of the specimen or slight errors in the fiber arrangement. To verify the derived DFOS displacement curves with an independent sensing technology, digital images were captured at each loading step and evaluated using Digital Image Correlation (DIC) to determine distributed displacements over the entire specimen length (Figure 7c). For detailed information on the DIC setup and its capabilities, see the work in [55]. The calculated differences between the displacement curves derived from DIC and DFOS in Figure 7d show an excellent agreement between OBR and DIC with deviations less than 0.5 mm, which is equivalent to an error of approximately 1%. Brillouin sensing techniques expose discrepancy’s origins at locations with curvature (=bending moment) changes, especially at the fixed bearing, which result in differences of more than 3 mm (≃ 7.5%). Additionally, the second change of the bending moment is overloaded by the BOTDR, which is why deviations to DIC at the floating bearing location at ~5.1 m are essentially higher compared to the other sensing technologies.

The outcomes suggest that all sensing techniques are capable to reproduce the deformation behavior based on the model-free shape sensing algorithm in civil engineering applications. However, the sensing technique and its capabilities regarding spatial resolution and measurement precision must be well selected with respect to requirements and the corresponding range of potential deviations must be taken into account within data interpretation. Additionally, it must be noted that the sensing range of the presented test setup is low compared to large scale civil infrastructure. For long-range applications, various supporting points can be used along the structure to identify and, finally, to control potential erroneous areas.

## 4. Concrete Beam Loading Tests

The interactive behavior of embedded sensors within the structure must be well understood before the installation to perform an appropriate data acquisition and interpretation. Depending on the sensor application method, the location of the sensor as well as the used sensing technology, divergent findings can be derived from fiber optic strain measurements, especially within an inhomogeneous structure consisting of concrete (cf. Figure 4). To understand the sensor’s behavior in a suitable manner, numerous concrete beams were instrumented using several sensor types and installation techniques like gluing of FiMT or tight-buffered fibers along the beam’s reinforcement, installation of sensing cables inside the concrete along the reinforcement, or gluing of tight-buffered fibers along the surface. The installations were monitored during vertical and bi-axial loading tests using DFOS sensing units based on Rayleigh and Brillouin scattering as well as by quasi-distributed fiber Bragg grating (FBG) interrogators.

**Figure 9:**
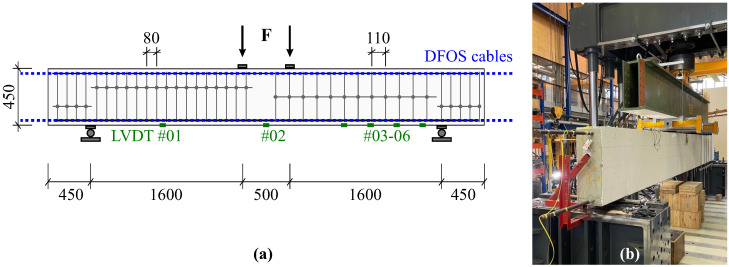
Laboratory beam testing: (**a**) Schematic representation of beam structure (dimensions in mm). (**b**) Instrumented concrete beam mounted at testing facility.

As an example, Figure 9 shows the schematic representation of one instrumented beam structure with a total length of 4.6 m and the corresponding setup at the testing facility. This test was carried out as a 4-point loading test, where the loading points are shifted 250 mm from the center, respectively. Besides the fiber optic sensing cables installed in two separate planes along the compression and tension reinforcement, the structure’s displacements were captured at six selected locations using Linear Variable Differential Transducers (LVDT).

### 4.1. Assessment of Different Sensor Installation Techniques

**Figure 10:**
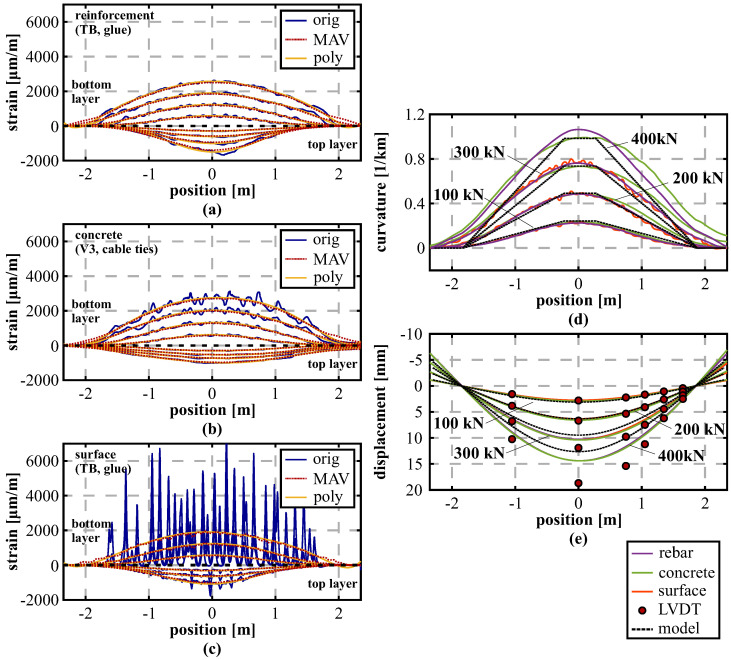
Analysis of different installation techniques: (**a**) Strain profiles along reinforcement (tight-buffered fiber, glue). (**b**) Strain profiles inside concrete (BRUsens V3, cable ties). (**c**) Strain profiles along surface (tight-buffered fiber, glue). (**d**) Curvature values derived from DFOS strains and theoretical model. (**e**) Displacement curves calculated from DFOS strains, measured by LVDT and theoretical model.

Within the first beam load test presented in this paper, the aim was to evaluate the impact of three different sensor application methods not only on the DFOS measurements, but also on the derived displacement curves. Figure 10a–c depicts the strain profiles measured by the OBR interrogation unit along the top and bottom sensing layer with a spatial resolution of 10 mm at four selected load steps from 100 to 400 kN (three load steps only for Figure 10c due to a fiber breakage). The strain profiles present an asymmetric behavior along both layers, which can be explained by the different amount of reinforcement with lower degree along the bottom compared to the top side of the beam. Furthermore, the outcomes strongly vary depending on the sensor location as well as the attaching technique. For instance, measurements along the tight-buffered fiber glued along the reinforcement bars (Figure 10a) show smoothed strain profiles with almost no irregularities, whereas the same fiber type glued along the surface (Figure 10c) depicts an heterogeneous behavior with numerous strain maxima. These strain peaks can be related to cracks, which are arising along the concrete’s surface with increasing load. Nevertheless, by applying appropriate low-pass filtering techniques like Moving AVerage (MAV) filter (here: filter length of 1 m) or polynomial filter (here: fourth-degree polynomial), it can be shown that the different sensor responses underlie the same structural behavior. This suggests that the strain-based shape sensing approach is applicable, even if the respective sensor output itself is essentially different. This knowledge also enables the possibility for subsequent installations in practical applications, where the sensor could not been installed during the construction.

The curvature profiles along the beam (Figure 10d) derived by relating the strain sensing layers of the different installation technique (here: MAV filtered) agree well to each other and also to the theoretical model, especially up to a load of 200 kN, where also the shape of the 4-point load setup can be well identified. Resulting from the test setup, it can be assumed that the beam’s supporting points at each side (1850 mm from the center) are stable to evaluate the distributed displacement curves along the beam depicted in Figure 10e. The results basically confirm the curvature profiles with good agreement to the LVDT sensors up to a load of 200 kN. At higher load steps, the displacement curves of different installation techniques match themselves, but significant deviations become visible, to the theoretical model, negatively, and to the LVDTs, positively. This leads to the assumption that the concrete beam can not fulfill the Bernoulli hypothesis of the consistent cross-sectional profile. This is why the strain-based shape determination algorithm is not capable to capture the actual displacement behavior after major cracking has arisen along the structure.

**Figure 11:**
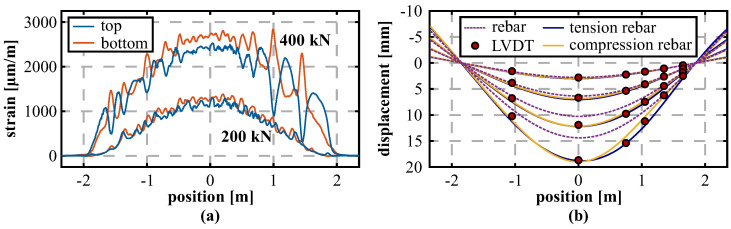
Optimized sensing approach along the reinforcement: (**a**) Strain profiles measured along two layers of tension reinforcement. (**b**) Derived displacement curves.

Nevertheless, also the theoretical model depicts essentially smaller displacements compared to the LVDT sensors, even with lower magnitude than the DFOS derivations. To investigate this conspicuous behavior in more detail, two respective sensing fibers were also installed in separate layers along the compression and tension reinforcement of the beam, which enables an analysis of the deformation behavior within the cross-sectional profile of the reinforcement bar without the concrete altering effect. Figure 11a shows the measured strain profiles along both instrumented layers of the tension reinforcement before and after the substantial cracking occurred along the beam. At a load step of 200 kN, the strain distributions depict a similar behavior with a slight offset in the middle area due to the vertical loading of the structure. With increasing load, however, not only the strain offset between the layers increases, but also significant bending becomes visible within the area between 1.0 and 1.5 m. This bending effect is resulting from a major shear crack, which arises due to the applied vertical loading and the lower degree of reinforcement at the right-hand side of the beam (cf. Figure 9). The measured data suggest that this crack causes a local buckling of the beam in vertical direction. Neither the theoretical beam model nor the strain-based DFOS derivation in different planes along the beam can cover this effect. Similar conclusions on the shear cracking impact were already drawn in literature [56]. The derived displacement curves in Figure 11b show that the actual deformation behavior of the beam, represented by the LVDT measurements, can be solidly captured even at higher load steps, when the determination is performed individually for each reinforcement layer.

**Figure 12:**
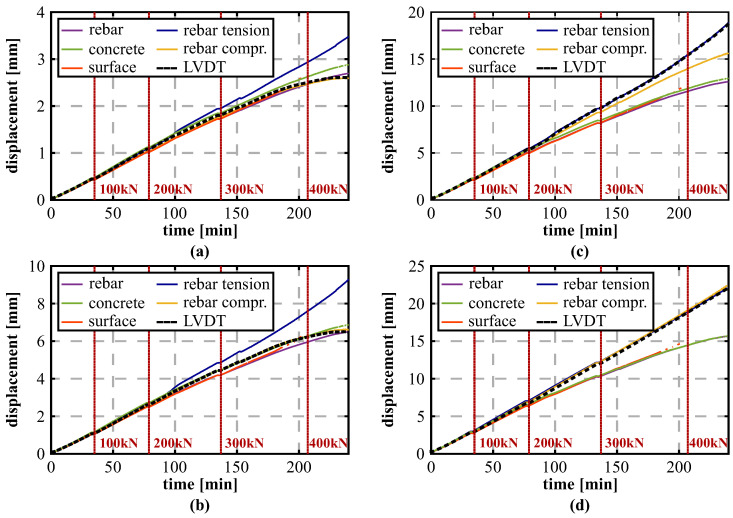
Comparison between DFOS and LVDT displacements over time: (**a**) LVDT #06 (+1.65 m). (**b**) LVDT #05 (+1.35 m). (**c**) LVDT #03 (+0.75 m). (**d**) LVDT #02 (0.00 m).

Between the load steps of 300 and 400 kN, slight deviations between the compression and tension reinforcement become visible along the shear cracked side of the beam. These can further be analyzed by comparing the deformation process for all different DFOS sensing approaches at the selected LVDT locations over time, see Figure 12 (displacement axis scale individually adjusted for each sensor position for better visibility). The results demonstrate that the beam basically follows two different states: Along the outside area at the locations of LVDT #05 and #06 (Figure 12a,b), the system depicts a nonlinear behavior at higher loads and reproduces the concrete behavior after cracking. Shape and magnitude of the deformations can also be captured within all DFOS derivations, except for the installation along the tension reinforcement. In contrast, the middle area of the beam at the positions of LVDT #02 and #03 (Figure 12c,d) displays an almost linear displacement increase and follows the deformation behavior of the reinforcement bar. This assumption can also be supported by the relative errors of the DFOS displacements with respect to the LVDTs derived at a load level of 300 kN (last load step before fiber breakage along surface installation), which are listed in Table 3.

Nonetheless, up to a load of about 220 kN, the DFOS displacements agree well to the LVDT sensors and represent the beam’s actual deformation behavior independent from the sensor location and its application. Here, the mean percentage error at the LVDT locations range from 2.9% (concrete) to 5.1% (surface), equal to a maximum absolute deviation of lower than 0.2 mm. All tested installation techniques seem therefore to be practicable to monitor distributed displacements along civil infrastructure objects within the usual working range before major cracking.

### 4.2. Assessment of Different Sensing Principles

As already discussed in Section 3, the spatial resolution of the interrogation technique majorly influences the outcome of the strain-based shape-sensing approach. This relation can be further analyzed for practical environments based on another concrete beam sample with a total length of 6 m, which was instrumented with the BRUsens V3 strain sensing cable in two layers. During the 4-point loading test, the sensors were interrogated by the OBR unit (Δz = 10 mm) as well as using a Brillouin Optical Frequency Domain Analyzer (BOFDA) from fibrisTerre (Germany) with a spatial resolution of 50 cm. Additionally to the DFOS cables, two FBG sensor chains (10 FBGs per chain) were attached along the bottom and top reinforcement layer of the beam, which also enables a direct comparison between quasi-distributed sensors and DFOS for model-free shape-sensing methods.

**Figure 13:**
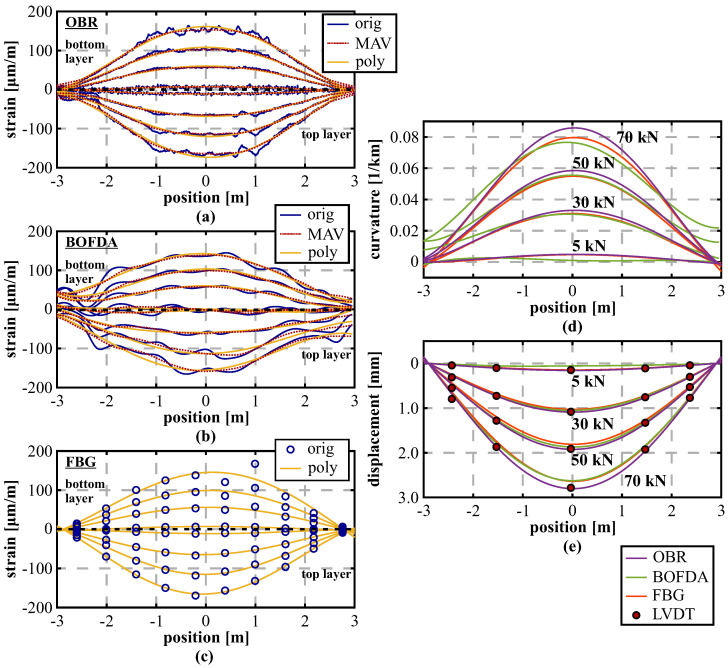
Analysis of different sensing units: (**a**) Distributed strains (OBR). (**b**) Distributed strains (BOFDA). (**c**) Quasi-distributed strains (FBG) (**d**) Derived curvature profiles. (**d**) Displacement curves calculated from DFOS strains and measured by LVDT. (**e**) The distributed displacement curves along the beam.

Figure 13a–c depicts the strain values measured by the different sensing technologies at four selected load steps (5 kN, 30 kN, 50 kN, 70 kN). For this example, lower load steps within the estimated elastic range of the beam were chosen to avoid major influences due to concrete cracking, which is not within the focus of this investigation. By comparing the strain profiles of the different DFOS sensing technologies (Figure 13a,b), it is obvious that the BOFDA technique can also solidly reproduce the shape and magnitude of the applied load, even if the OBR provides a higher quality with respect to spatial resolution and measurement precision. The installed FBG sensors deliver 10 pointwise strain readings along both layers, which can be distributed along the beam by, e.g., polynomial fitting (here: forth-degree polynomial). The resulting strain curves show a similar behavior compared to the DFOS distributions. While the polynomial fit also agrees well with the individual FBG values along the compression reinforcement, two sensing locations (+0.39 m and +0.99 m) along the tension reinforcement depict divergent readings. Contrary to a strict pointwise analysis, their impact on the strain curves is however limited due to polynomial fitting. The derived curvature profiles (Figure 13d) as well as the distributed displacement curves along the beam (Figure 13e) show that the beam’s vertical bending behavior can be clearly identified within all sensing approaches and the numerical displacements coincide well with the LVDT values.

**Figure 14:**
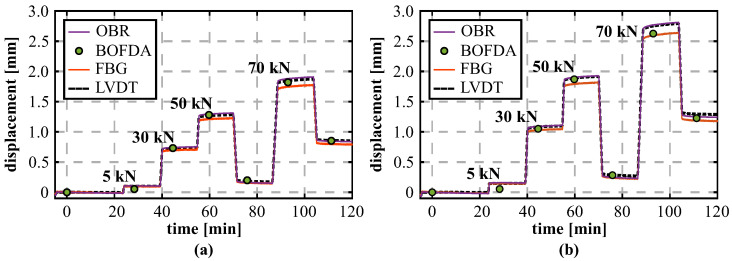
Comparison between DFOS and LVDT displacements over time: (**a**) LVDT left (−1.50 m). (**b**) LVDT center (+0.00 m).

The temporal order of the individual load steps, depicted for two selected LVDT locations over time in Figure 14, demonstrates that the individual loading sequences can be reliably identified, and even the smallest load level of 5 kN with a displacement change of 0.15 mm only can be well separated using the OBR as well as the FBG-based sensing approach. The measurement repeatability (1σ-level) of the different sensing principles, derived from subsequent measurements at a constant load of 30 kN, can be indicated with about 0.04 mm (BOFDA) and lower than 0.01 mm (OBR/FBG). The percentage errors between the LVDT sensors and the strain-based shape-sensing approaches at the highest load step of 70 kN in Table 4 show comparable deviations to the laboratory tests (OBR approximately 1%; BOFDA ≤ 5%) in Section 3, except for the LVDTs at the outside locations. It must be additionally noted that the individual shape sensing approaches (especially BOFDA and FBG) might be further optimized by eliminating single sensing points from the strain curve fitting, which is however not further pursued at this point.

**Figure 15:**
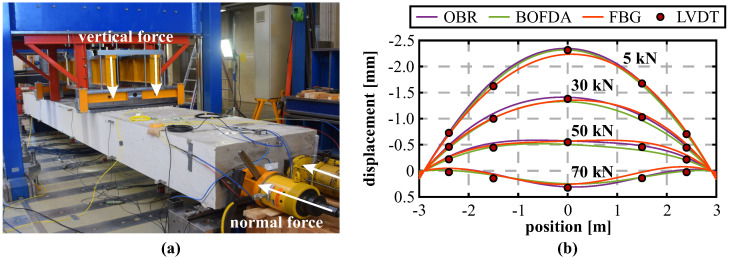
Combination of vertical loading and normal force: (**a**) Test setup. (**b**) Displacement curves derived from DFOS strains and measured by LVDT.

Besides the vertical loading, the investigated beam was also exposed to normal forces (see Figure 15a) to increase the loading capacity within the non-cracked state for further investigations (not shown in this paper). This bidirectional loading scenario of the beam sample enables an assessment of the strain-shape sensing method in the event of a force superposition. The resulting displacement curves in Figure 15b show that the normal force application (here: 1600 kN) results in a heave of the beam in the middle area, which is stepwise compensated by the vertical load introduction. The loading combination can be well reproduced by all sensing technologies and agree again to the LVDT displacements in shape and magnitude within the specified range.

## 5. Case Study: Precast Tunnel Lining Segments

**Figure 16:**
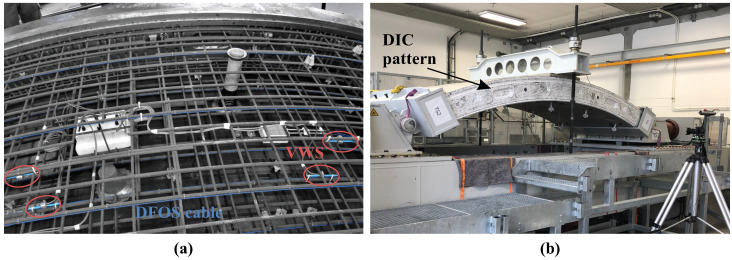
DFOS along precast tunnel lining segments: (**a**) Segment manufacturing. (**b**) Test rig.

When using tunnel boring machines (TBMs) for tunnel excavation works, it is state-of-the-art to apply precast concrete lining segments, which are usually set up in a ring of four to eight elements for support of the excavation cavity. IGMS in cooperation with the Chair of Subsurface Engineering (Montanuniversität Leoben, MUL) designed and developed a distributed fiber optic sensing approach to assess the distributed strain behavior inside tunnel lining segments [57]. The concept is based on one single sensing cable per segment, which is guided along the reinforcement or any supporting structure for steel fiber concrete segments [58]. This enables the realization of a sensing grid (Figure 16a) and, thus, a complete coverage of the tunnel segment with hundreds or even thousands of sensing points depending on the used measurement principle and the cable guiding. The fiber optic connectors are stored in connection boxes for protection during the concreting process. Inside the tunnel, the individual concrete segments can be connected to one continuous sensing loop to evaluate the ring’s overall behavior [59].

To verify the load-bearing and deformation behavior of precast tunnel lining segments under well-known loading conditions, the Austrian Federal Railways (OEBB Infrastructure AG) in cooperation with MUL developed and realized a special test rig (Figure 16b). The facility enables bi-axial testing of real-scale segments with different geometries and dimensions to optimize the segment design [60]. Additionally to two completed ring installations inside a railway tunnel in Austria, IGMS equipped eight tunnel segments with the designed DFOS approach to validate the system under controlled vertical, horizontal and bi-axial loading at the test rig [59,60,61]. The resulting strain sensing profiles along different layers of the segment can also be utilized to determine shape changes of a real-scale civil infrastructure object under load.

### 5.1. Shape Sensing Algorithm

**Figure 17:**
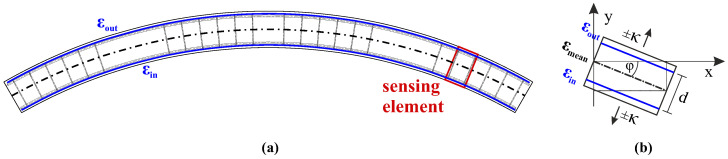
Shape sensing principle: (**a**) Scheme of tunnel segment cross section. (**b**) Detailed view of single sensing element.

Contrary to linear beam structures, tunnel lining segments present a curved initial state, which must be well considered within the shape-sensing algorithm. The sensing cable is guided along the outer and inner reinforcement (Figure 17a) of the tunnel segment with different distances to the center of curvature. This leads to slightly different lengths along both layers of the individual sensing elements, which can be taken into account by the different radii of the tunnel lining segment design [62].

The derived value of one individual sensing element (cf. Equation (2) to Equation (4)) represents the local curvature change due to stresses acting orthogonal to the tangent to the lining segment. By considering the initially curved geometry, the proportional curvature impact can be expressed within a two-dimensional coordinate system by

(5) $$\left[\begin{array}{c} \kappa^{x}\\ \kappa^{y} \end{array}\right]=\left[\begin{array}{c} \sin\varphi \\ \cos\varphi \end{array}\right]\cdot \kappa$$

where φ is the orientation of the sensing segment relative to horizontal coordinate axis *x* (Figure 17b). This geometry parameter can be initially retrieved from the tunnel segment design. The numerical integration process is performed individually for each coordinate direction. As the curvature values of each segment $\kappa^{x}$ and $\kappa^{y}$ are always related to the lining’s geometry, the double process integration can be understood as an iterative approach, where the lining’s geometry (φ) is continuously updated. The boundary-value problem is solved by adding additional observation of, e.g., LVDT sensors at the test rig or readings of total station measurements within the tunnel to define the absolute position and orientation. For closed ring system, it can be assumed that the displacement value and its gradient at the starting point is equivalent to the last integration position by extending the functional model with constraints instead of pointwise displacement readings. More detailed information on the functional model and the updating process is given in [52].

It is obvious that displacements in the plane of the x-axis are minimal for measurements at the test rig due to the loading orientation (cf. Figure 17b). Therefore, these are not further considered within this publication. When lining segments are however placed inside the tunnel, the orientation is essential, not only for the functionality of the shape sensing algorithm with respect to the supporting points, but also for the geotechnical interpretation of arsing deformations.

It is also remarkable that the torsion at the joints between the individual segments of the ring system cannot be captured by the DFOS approach. At these locations, additional displacement transducers could be used to support the double integration process and, finally, to provide a holistic view of the entire cross section.

### 5.2. Comparison to Conventional Sensors within Vertical Loading Test

**Figure 18:**
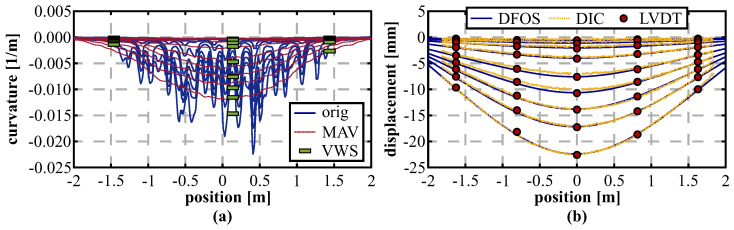
Vertical loading from 50 kN to 450 kN (steps of 50 kN): (**a**) Curvature profiles derived from DFOS strains and VWS readings. (**b**) Displacement curves calculated from DFOS strains and DIC compared to pointwise LVDT readings.

The first presented tunnel segment sample was subjected to one-axial loading in vertical direction, where the load was stepwise increased (50 kN to 450 kN in steps of 50 kN) and DFOS strain measurements were performed using the high-resolution OBR. The resulting curvature profiles displayed for one selected reinforcement layer in Figure 18a coincide with the general assumption of vertical bending due to the applied load. The curvature values derived from the original strain data along both layers however depict major irregularities. These can be related to cracks, which arise along the inner (tension) reinforcement of the segment. The curvature can be smoothed by filtering the original strains using a low-pass MAV filter (here: filter length of 1 m) to reduce the impact of local strain effects.

The utilization degree of tunnel lining segments is usually derived based on curvature values from strains measured by pairs of vibrating wire sensors (VWS) at specific locations along both reinforcement layers [63]. The sensors deliver strains within a gauge length of 15 cm and were also installed within the observed segments for verification purposes (Figure 16a). The resulting curvatures of three pairs of VWS sensors in Figure 18a basically agree with the values determined from the original DFOS data, but it seems that the derived value strongly depends on the sensor position itself. Especially in case of major local distortions like cracks, the utilization behavior might therefore be evaluated erroneously, although the local strains and curvature values are correctly determined. Here, the DFOS system can provide both local stress events as well as the overall assessment of the structural behavior by using appropriate filtering techniques.

Distributed displacement curves along the entire segment can be derived from the DFOS curvature values for each load step, see Figure 18b. The individual loading changes can be clearly identified and the numerical displacements agree well to the LVDT sensors in shape and magnitude, even if this behavior is partly implied by the correlation within the sensing algorithm. The percentage errors of the DFOS derivations in relation to the individual LVDT sensor locations basically decrease with increasing load (Table 5). Especially at lower load steps smaller than 200 kN, this behavior can be explained by the small deformation magnitudes, which result in significantly higher relative errors at lower loads. This relation was already demonstrated by DFOS investigations along concrete beams [64]. From 250 kN to 450 kN, the displacement deviations at the LVDT positions are within a range of 4% with maximum absolute deviations of 0.4 mm. Similar outcomes can also be captured by DIC reference measurements with maximum absolute deviations of smaller than 0.6 mm over the entire segment length. Especially at load steps starting from 350 kN, the completely independent measurement technique depict an excellent agreement with a percentage error between 1.3 and 1.7%, which additionally demonstrates that the designed DFOS approach is capable for real-scale civil infrastructure monitoring.

**Figure 19:**
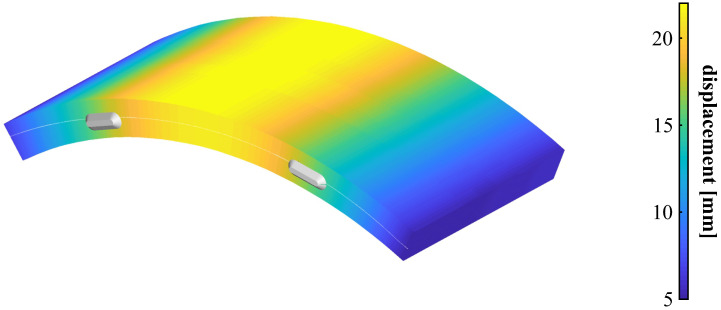
Areal displacement map of tunnel segment derived from DFOS at highest load step.

The tested tunnel segment was instrumented with six individual DFOS cable layers along both reinforcements to provide a complete coverage (cf. Figure 16a). Displacement curves can therefore be individually derived for each layer to form an areal sensing mesh with more than 2400 evaluation points. The combined areal displacement map in Figure 19 depict a uniform, symmetric deformation behavior along the entire segment. This is reasonable due to the consistent vertical loading, which is why the individual displacement curves depict similar results in shape and magnitude. Any combination of the vertical loading with stresses orthogonal to the segment’s cross section (i.e., in direction of the tunnel drive) would, however, result in torsion. In addition to locally strained sections and irregularities along the structure like cracks, such torsion effects could also be captured using the deployed DFOS approach.

### 5.3. Assessment of Different Sensing Principles

**Figure 20:**
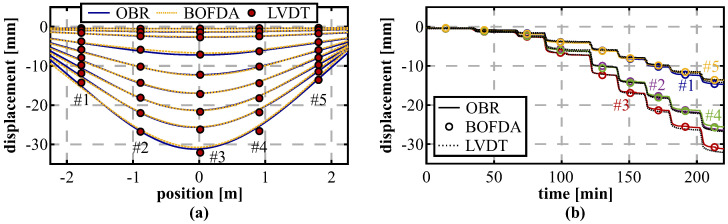
Analysis of different sensing techniques during vertical loading from 50 kN to 450 kN (steps of 50 kN): (**a**) Displacement curves calculated from OBR and BOFDA measurements compared to pointwise LVDT readings. (**b**) Displacements at LVDT locations over time.

To assess the spatial resolution impact of different interrogation techniques, the DFOS installation of another segment under vertical loading was alternately interrogated by the OBR as well as using the BOFDA sensing unit. Figure 20a proofs that the Brillouin interrogator can solidly capture the arsing deformation behavior with maximum deviations between the different DFOS technologies of 0.5 mm. By comparing the displacement values resulting from the DFOS techniques at the LVDT locations (Figure 20b), it becomes obvious that the loading process can be well identified and both technologies agree well to the displacements measured by the LVDT sensors over time. At the highest load step of 450 kN, deviations between the LVDTs and both technologies at the LVDT locations range from 0.6% to 3.9% with a mean percentage error of 2.4% (OBR) and 2.7% (BOFDA). The suitability of Brillouin sensing techniques, even with limitations in the spatial resolution, is essential for practical applications inside the tunnel, where the OBR’s restricted sensing range of 70 m can be disadvantageous in many cases.

### 5.4. Horizontal Loading Test

**Figure 21:**
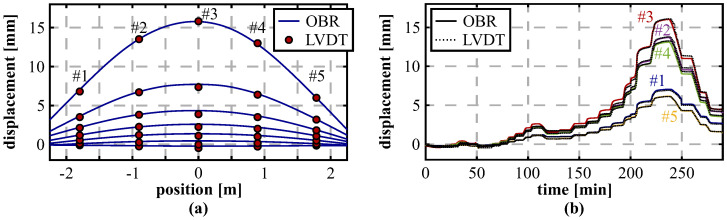
Horizontal loading from 100 kN to 700 kN (steps of 100 kN): (**a**) Displacement curves. (**b**) Displacements at LVDT locations over time.

Another lining segment was also investigated under horizontal loading at the test rig. This setup is assumed to result in vertical bending of the segment in upwards direction and, therefore, provides an additional loading scenario for the DFOS shape sensing approach. The displacement curves in Figure 21a reproduce these expectations and the derived displacements again agree well with the LVDT sensors. The mean percentage error of the DFOS measurements with respect to the LVDTs can be indicated with about 0.9% at the highest load step of 700 kN with a maximum absolute deviation of ~0.14 mm. Compared to the results of the vertical loading tests, this is even a slight numerical improvement and confirms the suitability of the designed shape sensing algorithm for curved structures.

The temporal deformation progress (Figure 21b) suggests that the horizontal loading is not performed fully symmetrically since the measured LVDT displacements at the left-hand side (#01 and #02) are slightly higher compared to corresponding ones on the other side (#04 and #05). Although the deviations between both sides are only in the range of 0.7 mm at the maximum load step, the derived DFOS displacements are definitely capable to identify this unsymmetrical behavior.

## 6. Discussion and Conclusions

This paper introduced a comprehensive analysis of a model-free strain-based shape-sensing concept along concrete structures. The approach is based on the double integration of distributed curvature values derived from fiber optic strain measurements along two layers in well-known arrangement along the structure. The capabilities of different sensor types, including numerous DFOS cables and FBG sensors, were evaluated within load tests of concrete beams as well as real-scale tunnel lining segments, where various installation techniques were used to attach the sensors along reinforcement, inside the concrete or at the structure’s surface. The installations were interrogated by fully-distributed strain sensing units based on Rayleigh and Brillouin scattering as well as by quasi-distributed FBG interrogators.

The results demonstrate that shape changes can be reliably determined using all installation techniques and sensing fibers before major cracking of the concrete arises. The strain sensing output can be, however, extremely different depending on the sensor type, the location as well as the attaching method, which must be taken into account for appropriate data interpretation. Evaluations of different DFOS technologies show that spatial resolution limitations or interpolation between FBG sensing points do indeed restrict the accuracy of the distributed shape sensing approach, but deviations are within the range of 5% or even lower for deformations larger than 1 mm. This can be particularly useful for practical applications, where sensing can not be performed using high-resolution interrogation units due to the range restrictions. The suitability of all derived displacement curves was proven not only by pointwise readings of LVDT sensors and theoretical model analysis, but also using completely independent verification measurements based on DIC.

**Figure 22:**
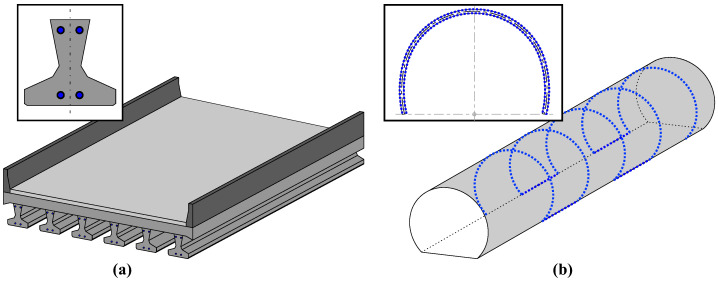
Seminal applications for distributed fiber optic shape sensing in civil structural health monitoring: (**a**) Bridge beams. (**b**) Tunnel cross sections.

The presented shape sensing design allows the model-free evaluation of distributed displacement profiles along the entire structure without a visual line-of-sight, which is not possible using conventional sensing techniques. Simultaneously, local distortions like cracks can be also identified using the same sensor, which is why the approach might be useful for numerous applications in civil structural health monitoring (CSHM) as exemplary shown in Figure 22. As a final remark, it must, however, be highlighted that the quality as well as the arrangement of the supporting points strongly influences the shape sensing algorithm. An appropriate design of all decisive sensing technologies is therefore essential to ensure the system’s performance in practical environment.

## Figures and Tables

**Table 1:** Characteristics of different distributed fiber optic strain sensing systems (specifications obtained from corresponding product datasheets [31,32,33,34,35,36,37,38,39] and laboratory tests, see, e.g., in [13]).

Scattering	Rayleigh		Brillouin	
Sensing technique	OFDR	TW-COTDR	BOTDA/ BOFDA	BOTDR/ BOFDR
Configuration	Single ended		Loop	Single Ended
Commercial manufacturers	Luna, Sensuron	Neubrex	fibrisTerre, Omnisens, Febus, OZ Optics, Neubrex	
Sensing range	70 m (2 km)	20 km	up to 80 km	up to 100 km
Spatial resolution	≤10 mm	2–20 cm	≥20 cm	≥100 cm
Typ. strain repeatability	≤1 μm/m	≤0.5 μm/m	≥2 μm/m	≥20 μm/m
Typ. measurement duration	<10 s	≤10 min	3–60 min	

**Table 2:** Derived spatial resolution impact lengths for different sensors and load levels.

Sensor Type	95% Strain Gradient Peak Width [cm]		
	at ∼500 με	at ∼1000 με	at ∼1500 με
SMF (Ormocer)	—	2.0	—
SMF 28e (Acrylat, clamped)	—	2.6	—
SMF 28e (Acrylat, glued)	11.2	11.3	12.0
Tight-buffered	8.5	8.1	8.3
BRUsens FiMT	1.8	1.9	1.9
BRUsens V9	10.2	10.0	10.3
BRUsens V3	15.4	15.5	15.6

**Table 3:** Percentage errors of different installation techniques at the 300 kN load step.

LVDT		Rel. Error [%]					
No.	Pos.	Rebar	Concrete	Surface	Rebar	Rebar Tension	Compr.
#01	−1.05 m	5.0	3.9	3.8	10.4	9.7	
#02	0.00 m	13.8	12.7	12.9	2.6	2.2	
#03	+0.75 m	16.1	13.2	15.7	0.0	4.3	
#04	+1.05 m	14.5	10.5	14.2	1.5	5.8	
#05	+1.35 m	5.5	0.1	5.3	9.6	0.2	
#06	+1.65 m	3.9	2.7	3.8	8.3	1.8	

**Table 4:** Percentage errors of different sensing units at load step of 70 kN.

LVDT		Rel. Error [%]		
No.	Pos.	OBR	BOFDA	FBG
#01	−2.40 m	9.4	9.3	15.2
#02	−1.50 m	1.0	1.1	4.9
#03	0.00 m	0.5	3.1	5.4
#04	+1.50 m	1.3	3.2	6.1
#05	+2.40 m	3.9	3.2	8.6

**Table 5:** Percentage errors for each load step during vertical loading at locations of LVDT sensors and mean percentage error with respect to distributed DIC measurements.

Load	Rel. Error [%]					
	#01	#02	#03	#04	#05	DIC
	−1.65 m	−0.85 m	0.00 m	+0.85 m	+1.65 m	—
50 kN	12.0	9.1	2.1	23.2	11.4	39.3
100 kN	9.2	3.0	7.5	47.6	9.4	26.4
150 kN	5.9	0.2	5.5	12.6	9.3	14.7
200 kN	3.1	0.1	2.5	3.0	5.3	5.7
250 kN	2.4	3.4	0.3	0.2	4.1	6.5
300 kN	2.1	3.2	0.8	1.5	3.9	4.8
350 kN	1.3	2.4	0.7	0.2	3.0	1.7
400 kN	0.8	2.2	0.6	0.6	2.5	1.4
450 kN	0.3	2.4	0.8	0.0	2.1	1.3

## Data Availability

Not applicable.
